# A meta-analysis and systematic review: adjuvant interferon therapy for patients with viral hepatitis-related hepatocellular carcinoma

**DOI:** 10.1186/1477-7819-11-240

**Published:** 2013-09-24

**Authors:** Shaojie Jiang, Yingxia Liu, Linhai Wang, Chenyang Duan, Mengying Liu

**Affiliations:** 1 Department of Hepatobiliary Surgery, Shenyang Military Region General Hospital, Shenhe District in Shenyang culture road no. 83, Shenyang 110015, China; 2 Department of Stomatology, Shenyang Military Region General Hospital, Shenyang, Liaoning 110015, China; 3 Company Five of Cadet Brigade, Third Military Medical University, Chongqing 400038, China; 4 Company Two of Cadet Brigade, Third Military Medical University, Chongqing 400038, China

**Keywords:** Interferon, Adjuvant treatment, Viral hepatitis, Hepatocellular carcinoma, Meta-analysis

## Abstract

**Objective:**

To evaluate the efficacy and safety of adjuvant IFN therapy for viral hepatitis-related hepatocellular carcinoma (HCC) after treatment with surgical resection or transarterial chemoembolization (TACE).

**Methods:**

Controlled trials of adjuvant treatment with IFN for patients with HCC published between 2000 and 2012 were searched electronically in MEDLINE, PubMed, Cochrane Library, and EMBASE databases. According to the heterogeneity of the studies, two different models - the fixed-effect model and the random-effect model - were applied to analyze the results.

**Results:**

Ten trials were screened according to inclusion and exclusion standards. Eight randomized, controlled trials and two non-randomized, controlled trials were included. These ten trials with a total of 1,029 subjects were eventually involved in the meta-analysis; 528 HCC patients were treated with adjuvant treatment with IFN and 501 patients with placebo. Compared to the control group, the recurrence rates of HCC in IFN group were significantly lower (odds ratio (OR) = 0.66; 95% confidence interval (CI) = 0.50 to 0.86; *P* = 0.02), especially after TACE treatment according to subgroup analysis (OR = 0.73; 95% CI = 0.52 to 1.01; *P* = 0.06 for surgical resection; and OR = 0.54; 95% CI = 0.33 to 0.86, *P* = 0.01 for TACE). The death rates in the IFN group also significantly decreased according to not only total events analysis (OR = 0.42; 95% CI = 0.32 to 0.56; *P* < 0.00001) but also subgroup analysis (OR = 0.51; 95% CI = 0.36 to 0.72; *P* = 0.0002 for surgical resection; and OR = 0.33; 95% CI = 0.21 to 0.50; *P* < 0.00001 for TACE).

**Conclusions:**

Adjuvant IFN therapy may significantly reduce the recurrence rates of patients with viral hepatitis-related HCC and improve the survival of patients after surgical resection or TACE. The ideal dose mostly selected is 3 MIU/ml, three times per week, which can make patients tolerate the adverse reactions of IFN better and maintain effective concentrations for a long time.

## Review

### Introduction

Primary liver cancer, which consists predominantly of hepatocellular carcinoma (HCC), is the fifth most common cancer worldwide and the third most common cause of cancer mortality [1]. Due to the high degree of malignancy and the difficulty of diagnosis in the early stage, the lesions of most patients have already metastasised when they first see a doctor [2]. However, only approximately 10 to 30% of patients may have the opportunity to receive radical therapies such as liver transplantation [3], but liver transplantation is only suitable for a small group of patients because of its high cost and strict selection criteria. Therefore, most patients receive surgical resection or transarterial chemoembolization (TACE) treatment [4]. Unfortunately, the recurrence rate of HCC 3 years after pure surgical resection or TACE is more than 50%, which is also the main cause of death after treatment [5]. As we know, most HCC patients carry the hepatitis B virus (HBV) and the hepatitis C virus (HCV). Since IFN can inhibit the replication of HBV and HCV and also kill the cancer cells, several researchers believe IFN may reduce the recurrence of HCC in patients after treatment [6,7]. But the conclusion is not consistent [6-8]. This paper raised clinical data through a Cochrane system evaluation, and used fixed/random effect models to perform a meta-analysis in order to study the therapeutic effects of radiofrequency ablation and hepatic resection in the treatment of small HCC, and provide a basis for evidence-based medicine.

## Methods

### Search strategy

#### Literature search

We reviewed all studies reporting the results of trials on the therapeutic effects of adjuvant treatment with IFN for patients with hepatocellular carcinoma (HCC). Computerized literature searches of MEDLINE, PubMed, Cochrane Library, and EMBASE databases, from 2000 to 2012, were undertaken. Search terms used were 'hepatocellular carcinoma', 'liver cancer', 'liver neoplasm', 'interferon', and 'IFN'. These terms were used in different combinations with each other. In addition, we reviewed the reference lists of the original articles and reviews on the topic to identify other possible eligible trials.

#### Study selection

The inclusion criteria for this meta-analysis were as follows: (1) all cases were diagnosed as HCC through pathology tests and combined with viral hepatitis (HBV/HCV); (2) both the IFN group and control group were treated with surgical resection or TACE; (3) adjuvant treatment with IFN lasted more than 3 months after the first treatment (surgical resection or TACE); (4) follow-up time was greater than 1 year, and the control group was treated with placebo; (5) the study subjects are all controlled clinical trial cases; (6) sample size greater than 20 cases.

The exclusion criteria for this meta-analysis were as follows: (1) metastatic hepatic carcinoma or recurrent liver cancers that have already been treated; (2) full text of the trial was not available; (3) when multiple publications from a single institution/author appeared to include duplication of patients, the study with smaller patient group was excluded; (4) follow-up time less than 1 year; (5) sample size ≤20 cases.

#### Data extraction

All literature searches were independently reviewed by two authors (CD and ML) to identify relevant trials that met the inclusion criteria and were checked by an independent reviewer. Disparities were resolved by discussion and mailed to the authors for explanation.

Included studies were reviewed based on the random fashion method, blind method and lost to follow-ups of the allocation plan. The grades to the creditability of the studies were divided into three grades according to Cochrane system evaluation handbook [9]: Grade A cases met all evaluated standards and had correct methodology, which gave a low risk of bias; Grade B cases did not describe one or several standards, which gave a moderate risk of bias; Grade C cases had one or several standards incorrect, which gave a high risk of bias. The methodology quality evaluations gave two studies as Grade A and eight studies as Grade B. Study quality was independently assessed by two reviewers (Table 1).

**Table 1:** Assessment quality of included studies

**Study**	**Trial type**	**Randomization**	**allocation concealment**	**Blinding**	**Lost of follow up**	**ITT**	**Grade**
Kubo S (2002) [9]	RCT	Adequate	Unclear	Adequate	Yes	Yes	B
Shiratori Y (2003) [10]	RCT	Adequate	Adequate	Adequate	Yes	Yes	A
Lin SM (2004) [11]	RCT	Unclear	Unclear	Unclear	Yes	Yes	B
Piao CY (2005) [12]	RCT	Adequate	Unclear	Adequate	Yes	Yes	B
Mazzaferro V (2006) [13]	RCT	Adequate	Unclear	Unclear	Yes	Yes	B
Sun HC (2006) [14]	RCT	Adequate	Adequate	Adequate	Yes	Yes	A
Lo CM (2007) [15]	RCT	Adequate	Adequate	Unclear	Yes	Yes	B
Li M (2009) [16]	RCT	Adequate	Unclear	Unclear	Yes	Yes	B
Li N (2010) [17]	NRCT	Adequate	Adequate	Unclear	Yes	Yes	B
Ishikawa (2012) [18]	NRCT	Adequate	Unclear	Unclear	Yes	Yes	B

### Data analysis

Statistical analyses were performed using Review Manager Software (RevMan 5.2; Cochrane Collaboration, Oxford, UK). Continuous descriptive data of the IFN group and the control group were reported as the mean ± standard deviation and dichotomous data were recorded as the case number (n).

The Mantel-Haenszel Q-statistic was used to assess heterogeneity among the studies and the I^2^ statistic was computed to examine the proportion of total variation in the study estimate due to heterogeneity. We considered *P* > 0.10 or *P* ≤ 0.10, *I*^2^ ≤ 50% to indicate no significant heterogeneity between the trials and selected a fixed-effect model to perform analysis. Furthermore, we considered *P* ≤ 0.10, *I*^2^ > 50% to indicate significant heterogeneity and used a random-effect model to perform analysis. The integration results regarded *P* < 0.05 as the standard for statistical significance.

Extensive effort was made to remove all duplicated data and to include all studies published to date. Publication bias in outcomes was assessed and treated using standard methodology. Funnel plots were used to visually inspect the relationship between sample size and treatment effects for the two groups.

## Results

### Search results

**Figure 1:**
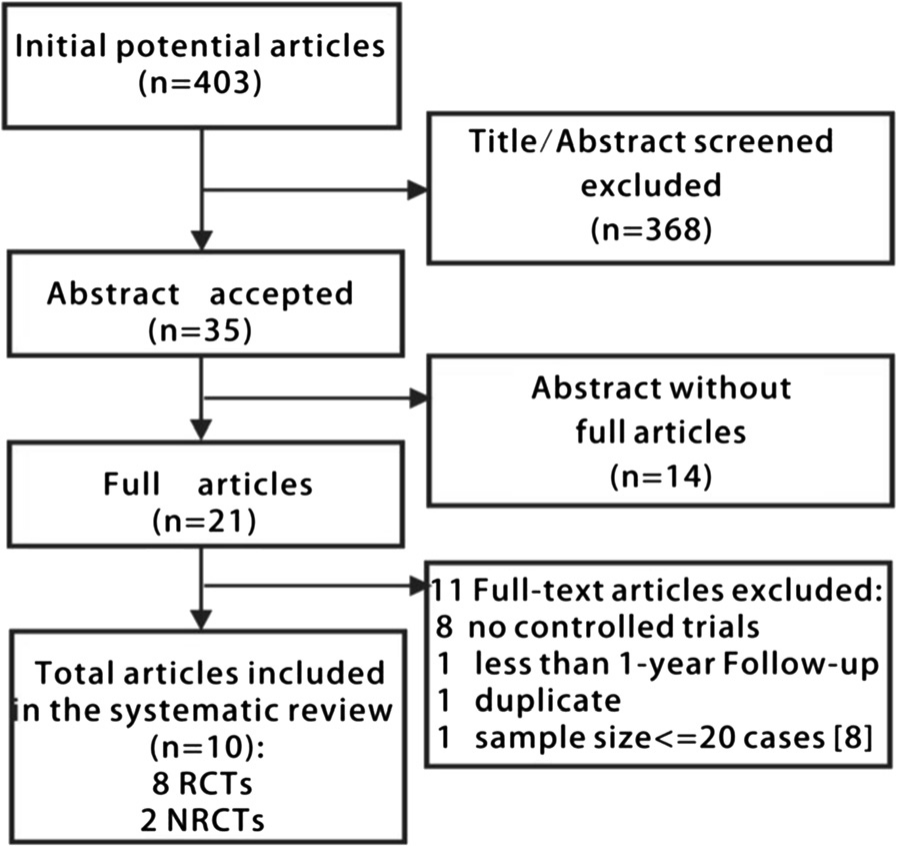
**Process of study selection.** The excluded 11 articles include eight articles without controlled trials, one article’s patients follow-up less than one year ,one article’s sample size <=20 cases and two articles from a single author appeared to include duplication of patients, only the study with the largest patient group was included and the other one was excluded.

A total of 403 relevant articles were identified in a combined search of MEDLINE, PubMed, Cochrane Library and EMBASE databases, from 2000 to 2012, and by a manual approach (search of studies cited in previous reviews and of reference lists from the identified articles); 368 articles were excluded because they were not relevant for the purpose of this meta-analysis through scanning the title/abstract. Full-length articles could not be retrieved for 14 of the 35 accepted articles for various reasons. Of the 21 full articles retrieved, 11 articles were excluded (see Figure 1 for the reasons). Eventually, 10 articles with a total of 1,029 subjects were included in the systematic review, including eight randomized, controlled trials and two non-randomized, controlled trials (Figure 1).

A total of 1,029 subjects were eventually included in the meta-analysis: 528 HCC patients were treated with adjuvant treatment with IFN and 501 patients with placebo. The longest follow-up time was 85.2 months and the shortest follow-up time was 24.0 months. The largest sample size was 236 and the smallest size was 30. The characteristics of the 10 studies included in the meta-analysis arre listed in Table 2 and the applications of adjuvant treatment with IFN in the IFN group are listed in Table 3.

**Table 2:** Characteristics of the studies included in the meta-analysis

**Study**	**Group**	**Cases**	**Average age (ys)**	**Male rate (%)**	**Tumor size (cm)**	**No. of tumor≥2 (%)**	**Cirrhosis (%)**	**Child-Pugh (%,A/B)**	**Hepatic virus**	**Therapy**
Kubo S (2002)	Interferon	15	61.9	100.0	2.5	0	46.7	73.3/26.7	HCV	Surgical resection
	Placebo	15	60.0	100.0	2.6	0	52.2	80.0/20.0		
Shiratori Y (2003)	Interferon	49	61.0	71.4	2.2	34.7	100.0	--	HCV	Surgical resection
	Placebo	25	63.0	68.0	2.3	36.0	100.0	--		
Lin SM (2004)	Interferon	20	61.5	80.0	2.2	15.0	90.0	85.0/15.0	HBV/ HCV	TACE
	Placebo	10	59.0	70.0	2.5	20.0	100.0	90.0/10.0		
Piao CY (2005)	Interferon	30	59	76.7	2.3	23.3	13.0	60.0/36.7	HBV/ HCV	Ablation/TACE/surgical resection
	Placebo	40	58	72.5	2.5	17.5	23.0	55.0/42.5		
Mazzaferro V (2006)	Interferon	76	65.0	80.3	3.4	22.4	94.0	92.1/7.9	HCV	Surgical resection
	Placebo	74	67.0	68.9	3.2	25.7	88.0	94.6/5.4		
Sun HC (2006)	Interferon	118	52.2	89.8	4.3	13.6	83.1	--	HBV	Surgical resection
	Placebo	118	50.4	86.4	4.9	12.7	88.1	--		
Lo CM (2007)	Interferon	40	49.0	77.5	5.5	17.5	47.5	--	HBV/ HCV	Surgical resection
	Placebo	40	54.0	85.0	5.7	27.5	47.5	--		
Li M (2009)	Interferon	108	50.3	71.3	4.9	54.6	100.0	90.7/9.3	HBV	TACE
	Placebo	108	49.2	68.5	4.8	54.6	100.0	91.7/8.3		
Li N (2010)	Interferon	43	53.2	72.5	3.2	37.2	88.0	85.0/15.0	HBV	TACE
	Placebo	36	51.2	71.3	3.4	37.2	88.0	85.0/15.0		
Ishikawa (2012)	Interferon	29	59.0	83.2	2.3	13.5	100.0	--	HCV	TACE
	Placebo	25	58.7	81.2	2.5	17.4	100.0	--		

Abbreviation: *TACE* trans-catheter arterial chemoembolization.

**Table 3:** Applications of adjuvant treatment with interferon in IFN group

**Study**	**Intervention**	**Follow-up (months)**
Ikeda K (2000)	10 patients received natural IFN-β 6×10^6^ twice a week for 36 months	25.0
Kubo S (2000)	15 patients received 6×10^6^ IFN-α intramuscularly every day for 2 weeks, then three times weekly for 14 weeks, and finally twice weekly for 88 weeks.	60.0
Shiratori Y (2003)	49 patients received 6×10^6^ IFN-α intramuscularly three times weekly for 48 weeks.	85.2
Lin SM (2004)	20 patients received 3×10^6^ IFN-α 2b intramuscularly three times weekly for 24 months.	27.0
Piao CY (2005)	30 patients orally received 100 mg of lamivudine daily after HCC treatment.	24.0
Mazzaferro V (2006)	76 patients received 3×10^6^ IFN-α 2b three times weekly for 48 weeks.	45.0
Sun HC (2006)	118 patients received 3×10^6^ IFN-α intramuscularly twice a week for 2 weeks and then 5×10^6^ three times weekly for 18 months.	36.5
Lo CM (2007)	40 patients received 10×10^6^/m^2^ IFN-α 2b subcutaneously three times weekly for 16 weeks.	30.0
Li M (2009)	108 patients received IFN-α 2b 3×10^6^ three times weekly by intramuscular injection one week.	24.8
Li N (2010)	43 patients received lamivudine with or without adefovir dipivoxil.	24.0
Ishikawa (2012)	29 patients received PEG-IFN α-2b/ribavirin after curative HCC treatment.	36.0

### Meta-analysis

We primarily compared recurrence rates and death rates for HCC between radiofrequency ablation (RFA) and hepatic resection (HR) in the treatment of small HCC. In order to further detect if there are any difference in recurrence rates and death rates based on the different treatment types, we performed subgroup analysis based on the treatment type (surgical resection/TACE). The results of meta-analysis for each indicator are listed below.

#### Recurrence rates for hepatocellular carcinoma

Nine studies including 975 cases [9-17] compared the IFN group and the control group with regard to the recurrence rates of HCC. Taking the odds ratio (OR) as an indicator of the effect of measures, we performed a χ^2^ test to examine the heterogeneity; the result was *P* = 0.67 (>0.10), I^2^ = 0% (<50%), which indicated that there was no heterogeneity between the two groups. Therefore, we used a fixed-effect model to perform a meta-analysis. The results (OR = 0.66; 95% confidence interval (CI) = 0.50 to 0.86, *P* = 0.02) indicated that adjuvant treatment with IFN might significantly decrease the recurrence rates of HCC after initial treatment.

**Figure 2:**
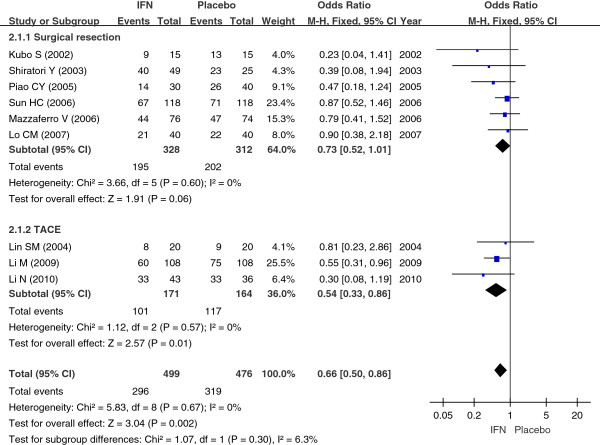
**The comparison between IFN group and control group in the recurrence rates of HCC.** Abbreviation: IFN- interferon; TACE- transarterial chemoembolization. Summary ORs are shown as diamonds, with the middle corresponding to the point estimate and the width representing the 95% CI.

In order to further reinforce the conclusion above, we performed subgroup analysis based on the treatment type (surgical resection/TACE). We performed a χ^2^ test to examine the heterogeneity; the results were *P* = 0.60 (>0.10), I^2^ = 0% (<50%) and *P* = 0.57 (>0.10), I^2^ = 0% (<50%), respectively, which indicated that there was no heterogeneity. Therefore, we used a fixed-effect model. The results (OR = 0.73; 95% CI = 0.52 to 1.01; *P* = 0.06 for surgical resection; and OR = 0.54; 95% CI = 0.33 to 0.86; *P* = 0.01 for TACE) indicated that there was a significant difference between the IFN group after TACE treatment and the control group for recurrence of HCC, and there was no difference between the IFN group after surgical resection and the control group (Figure 2).

#### Death rates for hepatocellular carcinoma

Nine studies including 949 cases [9-11,13-18] compared the IFN group and the control group with regard to the death rates of HCC. Taking OR as an indicator of the effect of measures, we performed a χ^2^ test to examine the heterogeneity; the result was *P* = 0.54 (>0.10), I^2^ = 0% (<50%), which indicated that there was no heterogeneity between the two groups. Therefore, we used a fixed-effect model to perform a meta-analysis. The results (OR = 0.42; 95% CI = 0.32 to 0.56, *P* < 0.00001) indicated that adjuvant treatment with IFN might significantly decrease the death rates of HCC after initial treatment.

**Figure 3:**
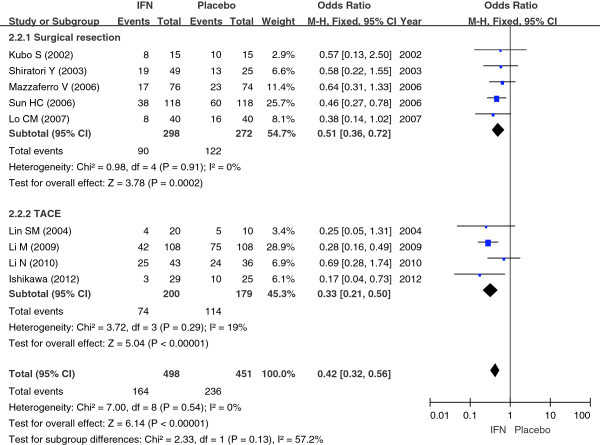
**The comparison between IFN and control group in the death rates of HCC.** Abbreviation: IFN- interferon; TACE- transarterial chemoembolization. Summary ORs are shown as diamonds, with the middle corresponding to the point estimate and the width representing the 95% CI.

In order to further reinforce the conclusion above, we performed subgroup analysis based on the treatment type (surgical resection/TACE). We performed a χ^2^ test to examine the heterogeneity; the results were *P* = 0.91 (>0.10), I^2^ = 0% (<50%) and *P* = 0.29 (>0.10), I^2^ = 19% (<50%), respectively, which indicated that there was no heterogeneity. Therefore, we used a fixed-effect model. The results (OR = 0.51; 95% CI = 0.36 to 0.72; *P* = 0.0002 for surgical resection; and OR = 0.33; 95% CI = 0.21 to 0.50; *P* < 0.00001 for TACE) indicated that there was a significant difference between the IFN group and the control group whether after surgical resection or TACE treatment in the death rates of HCC (Figure 3).

#### Sensitivity analysis and publication bias

**Figure 4:**
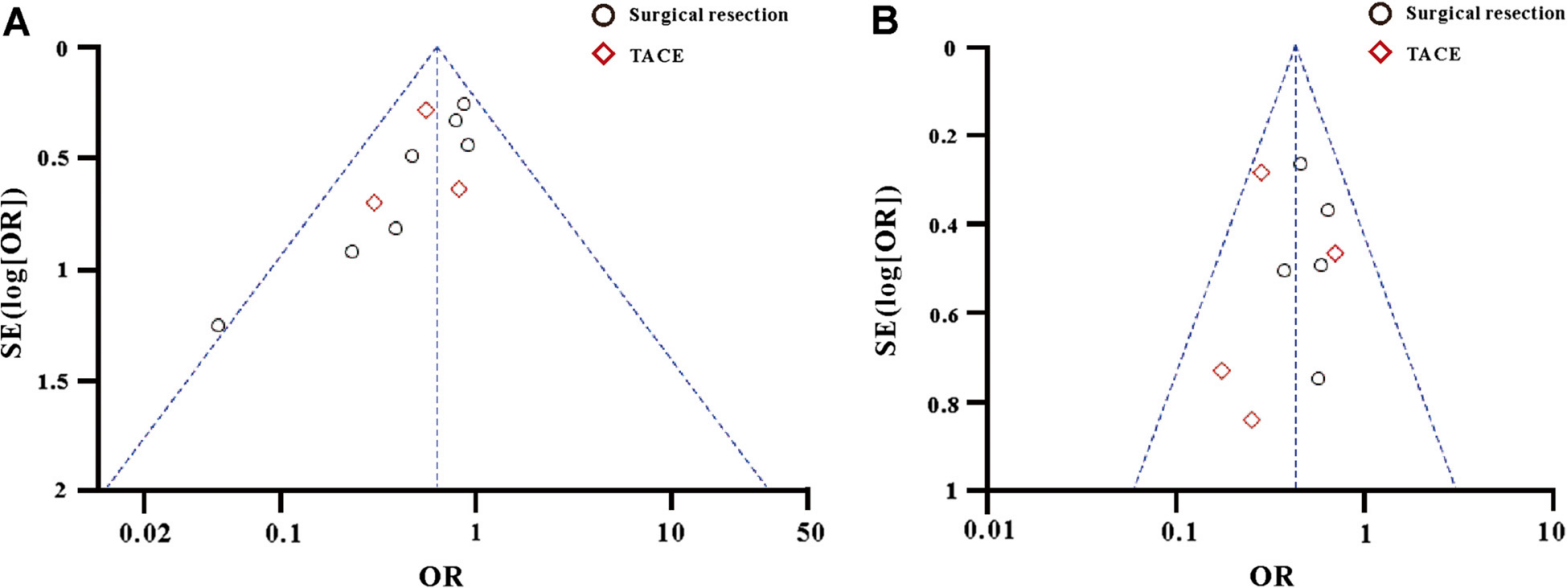
**Funnel plot for each comparisons. A**. Recurrence rate of HCC; **B**. Death rates of HCC; black circle represented surgical resection; red square represented TACE treatment for HCC.

We used fixed/random-effect models to test each indicator and the results were correlated with each other. We made a funnel plot for each comparison (Figure 4), including the subgroup comparisons. The characters of both plots were basically inverted funnel-shaped, with bilateral symmetry, which indicated that there was no publication bias and the conclusions were reliable.

## Discussion

This meta-analysis evaluates the efficacy and safety of adjuvant IFN therapy for viral hepatitis-related HCC after treatment by surgical resection or TACE. All cases were combined with viral hepatitis (HBV/HCV). The results of the meta-analysis showed that there was a significant difference between the IFN group and the control group both in the recurrence rates and death rates of HCC. In our clinical work, the main treatment method for HCC is surgery and TACE [19]. In order to exclude bias caused by treatment methods, we selected HCC treated by surgery and TACE rather than other curative treatments. We performed subanalysis between surgical resection and TACE to ascertain any difference between these two methods.

Only two included studies reported the adverse events of adjuvant IFN therapy [11,15]. The major adverse events were high fever, tiredness, a decrease in white cells and platelets, hepatotoxicity and hyperthyroidism, and so forth. The adverse events of IFN depend on the dose. Randomized, controlled trials reported by Lo and colleagues [15] were divided into a low-dose IFN group, a high-dose IFN group, and a control group. All the high-dose group (30 MIU/ml, three times per week) patients withdrew because of adverse events. The study by Sakaguchi and colleagues [20] also showed that long-term low-dose adjuvant IFN therapy could improve patient tolerance, which may improve the prognosis of treatment.

IFN has a variety of biological functions, such as anti-virus, resistance to cell proliferation, anti-angiogenesis and immune adjustment, and so forth, and has been widely applied in many kinds of diseases [21,22]. Experiments *in vitro*[23] showed that IFN-α could restrain the proliferation of human umbilical vein endothelial cells and its inhibition function was enhanced as the dose and action time were extended. Experiments *in vivo*[24] showed that the tumor diameter reduced significantly after adjuvant IFN-α treatment and microvascular density was significantly lower than in the control group. We can speculate that IFN-α realizes its anti-tumor effects by inhibiting tumor angiogenesis and resisting cell proliferation. The biological characteristics above may explain the benefit of adjuvant IFN therapy on HCC patients.

Many researchers have found that the efficacy of IFN on inducing Hepatitis Be Antigen (HBeAg) seroconversion is far from satisfactory [25], but we should realize that IFN acted mainly as an antiviral, immunomodulatory and antiproliferative agent and not as an inhibitor of the integration of HBV DNA [26]. IFN therapy improves overall survival through relieving active hepatitis, improving hepatic fibrosis and liver function [27] as well as decreasing the severity of a recurrent tumor and thus be amenable to secondary curative ablation or resection [28]. From the above, we can conclude that, although IFN does not prevent recurrence in HBV-related HCC patients, it still shows long-term beneficial effects in HBV-related HCC in other ways.

This study has the following limitations: (1) although most of the included studies are randomized, controlled trials, the sample sizes are relatively small; (2) the basic characteristics of included cases are not all the same, and the effect factors to prognosis of HCC include clinical stage, pathological type, treatment methods, the size of tumor and the body’s immune ability, which may also affect the results of meta-analysis; (3) the included studies report mostly on the recurrence rate and death rate; there are less reports on overall survival and disease-free survival of adjuvant IFN therapy to HCC.

## Conclusion

In conclusion, the meta-analysis based on 10 included studies indicates that adjuvant IFN therapy may significantly reduce the recurrence rates of patients with viral hepatitis-related HCC and improve the survival of patients after the treatment of surgical resection or TACE. The ideal dose mostly selected is 3 MIU/ml, three times per week, which can make patients tolerate the adverse reactions of IFN better and maintain an effective concentration for a long time because of IFN dose dependence.

## Abbreviations

CI: Confidence interval; HBV: Hepatitis B virus; HCC: Hepatocellular carcinoma; HCV: Hepatitis C virus; IFN: Interferon; OR: Odds ratio; TACE: Transarterial chemoembolization.

## Competing interests

The authors declare that they have no competing interests.

## Authors’ contributions

SJ independently searched references and extracted the parameters from each study, took charge of data statistics and drafted the manuscript. YL independently searched references and extracted the parameters from each study. LW participated in the discussion of the discrepancies between the two reviewers. CD independently searched references and extracted the parameters from each study and revised the manuscript. ML participated in the manuscript revision. All authors read and approved the final manuscript.
